# 非小细胞肺癌免疫治疗进展

**DOI:** 10.3779/j.issn.1009-3419.2014.01.06

**Published:** 2014-01-20

**Affiliations:** 100730 北京，中国医学科学院，北京协和医学院，北京协和医院呼吸内科 Department of Respiratory Disease, Peking Union Medical College Hospital, Chinese Academy of Medical Science and Peking Union Medical College, Beijing 100730, China

**Keywords:** 肺肿瘤, 免疫治疗, 肿瘤疫苗, 调定点阻滞剂, Lung neoplasms, Immunotherapy, Tumour vaccines, Checkpoint inhibitors

## Abstract

近年来，非小细胞肺癌（non-small cell lung cancer, NSCLC）在手术治疗、放疗、化疗及靶向治疗方面均取得了很大的进展，但晚期NSCLC患者的5年生存率仍然很低。免疫治疗利用免疫系统来控制和清除瘤细胞，已成为肿瘤治疗的一种重要手段。NSCLC的免疫治疗近期取得了突破性的进展，抗原特异性肿瘤疫苗、检查点阻滞剂等多种新型抗肿瘤免疫治疗药物已进行NSCLC治疗的临床试验，并在Ⅱ期和Ⅲ期临床试验中取得一定的成果。目前已完善了免疫治疗疗效评估标准，成为免疫治疗药物抗肿瘤评价标准。免疫治疗将成为NSCLC治疗的一种重要手段。

## Abstract (en)

In recent years, the five-year survival rate of patients with advanced stage non-small cell lung cancer (NSCLC) remains low despite recent advances in surgery, irradiation, chemotherapy, and targeted therapy. Immunotherapy which utilizes the immune system to control and eradicate cancer is a viable treatment approach for malignancy. Immunotherapy in patients with lung cancer has made breakthrough progress recently. Novel immunotherapeutic agents, such as antigen-specific tumour vaccines, checkpoint inhibitors, etc, have all been evaluated in lung cancer, and some have shown prolonged survival time in phase Ⅱ trials and Ⅲ trails. The immune-related response criteria for the evaluation of antitumor responses with immunotherapeutic agents have been made. Now, immunotherapy will likely be a fundamentally new concept for the treatment of NSCLC.

肺癌是男性患者发病率第二的肿瘤，也是男性肿瘤患者致死率最高的肿瘤^[1]^，约80%的肺癌患者是非小细胞肺癌（non-small cell lung cancer, NSCLC）。近几十年来，对于NSCLC的治疗取得了很大的进展，除了手术、放疗、化疗等基本治疗手段外，又出现了靶向治疗、免疫治疗等新兴疗法。生物靶向治疗以其针对性强、副作用小等优势，逐渐走上肿瘤治疗的舞台，酪氨酸激酶受体拮抗剂的广泛应用使得部分表皮生长因子受体突变的NSCLC患者临床获益^[2]^。然而，晚期NSCLC患者的5年生存率仍然很低。由于在肿瘤形成的过程中，人体自身的免疫异常起了很大的负性作用，因此，肿瘤的免疫治疗在很早就被认识和提出。随着免疫学和分子生物学的迅速发展，人们对肿瘤抗原及其呈递过程、肿瘤免疫耐受等问题有了新的认识和了解，肿瘤免疫治疗得到迅速发展，由于其针对性强、副作用小等优势受到人们的重视^[3]^。虽然肺癌是不典型的免疫原性的恶性肿瘤，但越来越多的证据表明，抗肿瘤细胞的免疫应答与肺癌患者的预后相关。近年来，肺癌的免疫治疗取得了突破性的进展，免疫治疗已成为肺癌新的治疗方法^[4, 5]^。此外，研究发现，看似矛盾的化疗和免疫治疗有协同增强作用，并由此产生了化学免疫治疗（chemo-immunotherapy），希望能够通过两种治疗的组合，进一步增强抗肿瘤治疗效果^[6, 7]^。因此，肺癌患者的免疫治疗越来越受到重视。

## 1 肿瘤的免疫逃逸

### 1.1 机体正常免疫监视

为了更好地了解肺癌的免疫治疗，首先应了解肿瘤发生与免疫系统的关系^[8]^。在肿瘤细胞产生的早期，因为肿瘤表达特异性的抗原，免疫系统可以识别肿瘤细胞为异常细胞。抗原呈递细胞（antigen-presenting cells, APCs），如树突状细胞（dendric cell, DC）和巨噬细胞，这些细胞加工肿瘤抗原，并联合主要组织相容性复合体（major histocompatibility complex, MHC）-1和2将他们在APC表面表达，不同的免疫效应细胞就会被激活，启动一些免疫过程，可以导致肿瘤细胞的凋亡，进而产生有效免疫监视，避免肿瘤发生。然而，当突变细胞逃脱机体免疫系统的监视和清除时，就会在体内分裂增殖进而形成肿瘤。影响肿瘤逃脱机体免疫监视的因素有很多，主要包括免疫抑制和免疫耐受。

### 1.2 肿瘤的免疫抑制

肿瘤的免疫抑制包括肿瘤诱导产生免疫抑制细胞和免疫抑制细胞因子^[9]^。肿瘤诱导产生免疫抑制细胞，如CD4^+^CD25^+^调节T细胞（regulatory T cells, Treg）和髓系来源抑制细胞（myeloid derived suppressor cells, MDSCs），最为常见。Treg是由CD4^+^CD25^+^T淋巴细胞的亚群在胸腺产生，在外周诱发，旨在保持内环境稳定和免疫沉默，是一个重要的自身核对。在人类，这些细胞占总的外周T细胞的2%-5%。Treg可以分泌免疫抑制因子，阻断效应T细胞功能和DC细胞向APC的转化。在肿瘤患者中，Treg获得生长优势，他们的促肿瘤作用是通过抑制T细胞功能和分泌免疫抑制因子如白介素10（interleukin-10, IL-10）和转化生长因子β（transforming growth factor, TGF-β）完成。NSCLC患者Treg在肿瘤组织中和外周血细胞中均有增高，这种Treg的增高促进了肿瘤增长。MDSCs是一种骨髓细胞来源的群体，可以逐渐地在肿瘤患者体内蓄积，并抑制T细胞介导的免疫反应。免疫抑制细胞因子包括TGF-β、IL-10等，其中TGF-β可以阻断T和B细胞的活性、NK和淋巴因子激活杀伤细胞和抗原呈递。

### 1.3 肿瘤免疫耐受

肿瘤免疫耐受的原因较多而复杂，主要原因可能是由于肿瘤细胞缺乏一种或多种成分，导致其免疫原性低下，而这些成分是有效刺激机体免疫系统所必须的。主要包括以下几点^[3, 10]^：①肿瘤细胞的免疫原性弱；②肿瘤抗原的封闭、遮蔽与隔离；③MHC分子的低表达：改变的MHC-1是肿瘤免疫逃避的主要机制之一，多种肿瘤中观察到此现象，且MHC-1表型阳性或阴性与肿瘤的免疫原性相关^[11]^；④共刺激因子缺乏；⑤抗原呈递障碍，如肿瘤可以通过妨碍Toll样受体（Toll-like receptors, TLR）配体介导的DC的分化成熟^[12]^；⑥肿瘤细胞免疫豁免，某些肿瘤细胞还可以通过营造局部免疫豁免以逃避免疫监视。此外，肿瘤可表达特殊的细胞表面分子，如程序死亡分子1配体（programmed death ligand 1, PD-L1），可以绑定免疫细胞相应受体，诱发免疫功能细胞死亡和失能。

## 2 肺癌的免疫治疗

尽管早期的免疫治疗方法，如IL-2、干扰素和第一代疫苗治疗效果欠佳，但新的免疫治疗方法有着巨大的进展。目前肿瘤的免疫治疗主要分为两大类：经典免疫治疗策略和免疫靶向治疗。

### 2.1 经典免疫治疗策略

经典免疫治疗策略通过多种策略增强免疫系统进行肿瘤治疗。一般通过3个策略：主动免疫治疗、被动免疫治疗和支持性免疫治疗。

#### 2.1.1 主动免疫治疗（肿瘤疫苗策略）

主动免疫治疗是指特异性的启动机体免疫系统识别肿瘤，增加抗肿瘤淋巴细胞，转化免疫抑制环境成为免疫刺激环境。这个战略主要是通过注射肿瘤疫苗来实现。肿瘤疫苗注射入肿瘤患者体内，诱发出潜在的CD4^+^和CD8^+^效应T细胞，清除肿瘤细胞。同时，肿瘤疫苗是肿瘤特异性的，以避免宿主细胞被特异性的效应T细胞攻击。

近年来，肺癌的肿瘤疫苗治疗取得了巨大的进展^[13]^，目前已发展多种肿瘤疫苗，包括抗原特异性的肿瘤疫苗，肿瘤细胞疫苗和DC细胞疫苗，一些已进入Ⅲ期临床试验。

##### 2.1.1.1 抗原特异性肿瘤疫苗（melanoma-associated antigen-A3, MAGE-A3）

MAGE-A3在多种肿瘤表达，但不表达于正常的组织（睾丸除外）。MAGE-A3在35%-55%的NSCLC患者中表达，是独立的肺腺癌预后差的预测因子^[14]^。目前MAGE-A3抗原特异性肿瘤疫苗是重组融合蛋白（MAGE-A3和流感嗜血杆菌蛋白D）联合免疫增强佐剂（ASO2b和AS15）疫苗。因为术后患者肿瘤负荷小，疫苗可以发挥最大作用，目前该疫苗也是唯一的肺癌术后治疗疫苗。

该疫苗Ⅱ期临床试验^[15]^主要针对Ib期和Ⅱ期行肺癌根治术的NSCLC患者，经检测MAGE-A3为阳性的患者纳入试验，共182例患者入组。NSCLC患者随机给予MAGE-A3疫苗或安慰剂（27个月共给予13个治疗剂量），不行辅助化疗，以无疾病间期（disease-free interval, DFI）为第一试验终点。经过中位术后时间44个月的随诊，MAGE-A3疫苗组肿瘤复发率是35%，而对照组是43%。在MAGE-A3治疗组与对照组相比，DFI[危险比（hazard ratio, HR）=0.75，95%CI: 0.46-1.23，*P*=0.254）]、无疾病生存期（disease-free survival, DFS）（HR=0.76, 95%CI: 0.48-1.21, *P*=0.248）和总生存期（overall survival, OS）（HR=0.81, 95%CI: 0.47-1.40, *P*=0.454），均无统计学差异，随诊延长至70个月，DFI及DFS结果与40个月相类似。这个治疗耐受性好，未观察到明显的毒副反应。基于以上结果，MAGE-A3疫苗的Ⅲ期临床试验MAGERIT（MAGE-A3 as adjuvant non-small cell lung cancer immuno therapy）正在进行，计划入组2, 270例行肺癌根治术的NSCLC，且没有接受标准术后化疗的患者，以进一步评估MAGE-A3疫苗的术后辅助治疗效果。

##### 2.1.1.2 L-BLP25

MUC1（mucinous glycoprotein-1）是高糖基化的跨膜蛋白，在多种肿瘤表达，功能尚未明确，可能与促进细胞生长和存活相关，在肿瘤细胞，MUC1失去极性表达，常常是过表达，有异常糖基化，导致肽表位暴露，因此是免疫治疗的潜能靶点^[16]^。L-BLP25疫苗是一个肽抗原为基础的疫苗，靶目标是针对暴露的MUC-1抗原的肽核心。L-BLP25疫苗包括BLP25脂肽（包含25个氨基酸）、可以促进APC摄取的脂质体呈递系统（包括胆固醇、磷脂酰甘油和二棕榈酰磷脂酰胆碱），以及可以帮助增强免疫刺激的单磷酰脂质A。

在Ⅱ期临床实验中，171例Ⅲb期和Ⅳ期NSCLC患者，经过一线化疗后已经得到疾病控制，随机给予L-BLP25（*n*=88）和最佳支持治疗（best supportive care, BSC）（*n*=83），给予L-BLP25的患者接受环磷酰胺（Cyclophosphamide, CTX）（300 mg/m^2^）3天，继而给予8周皮下注射L-BIP25疫苗，然后每6周1次，终点是OS。结果显示，L-BLP25组中位OS是17.4个月，对照组13个月（HR=0.739, 95%CI: 0.509-1.073, *P*=0.112）。分层分析发现，Ⅲb期有恶性胸腔积液和Ⅳ期患者没有生存优势，对于无胸腔积液的Ⅲb期患者，中位OS是未达：13个月（HR=0.524, 95%CI: 0.261-1.052, *P*=0.069）^[17]^。L-BLP25疫苗安全性好，副作用多数是I级流感样症状和注射部位反应。长期分析确定了两个发现：L-BLP25治疗组患者3年生存率提高，3年生存率治疗组是31%，对照组是17%（*P*=0.035）。对于局限的Ⅲb期患者，中位生存时间明显延长，为30.6个月，对照组是13.3个月（HR=0.548, 95%CI: 0.301-0.999）。对于该亚组，3年生存率是治疗组和对照组分别是49%和27%（HR=0.548, 95%CI: 0.301-0.999, *P*=0.070）^[18]^。基于上述的结果，Ⅲ期临床试验从2006年开始进行，包括1, 464例未切除的Ⅲ期NSCLC患者经过放化疗稳定或是有反应的患者，患者接受L-BLP25及BSC或安慰剂+BSC，终点是OS。

##### 2.1.1.3 TG4010

TG4010是一个重组的病毒疫苗，载体是修改后的Ankara病毒，修改后不仅表达肿瘤相关性抗原MUC1抗原也表达IL2。外源性注射IL-2是一个强烈的免疫佐剂，可以逆转肿瘤相关的MUC1粘液素导致的T细胞的抑制。

在一个多中心、单臂的Ⅱ期随机研究中^[19]^，148例未治疗的MUC1阳性的Ⅲb期及Ⅳ期患者接受6周期GP一线化疗，联合（*n*=74）或不联合TG4010（*n*=74）治疗，TG4010疫苗每周皮下注射6周，之后3周1次，第一终点是6个月无疾病进展时间（progression-free survival, PFS）。TG4010治疗组6个月PFS大于40%（治疗组43.2% *vs*对照组35.1%，*P*=0.13），TG4010治疗组有效率高于对照组（治疗组43% *vs*对照组27%，*P*=0.03）。亚组分析显示，有正常水平的激活NK细胞的患者，6个月PFS可达58%（*P*=0.04），该亚组OS可达18个月（*P*=0.020）。基于上述结果，一个Ⅱb期、Ⅲ期随机对照双盲试验正在进行，拟纳入1, 000例MUC1表达的有正常水平的激活NK细胞的Ⅳ期患者，该试验会于2011年结束。

##### 2.1.1.4 NY-ESO-1（NewYork esophageal squamous cell carcinoma-1）抗原特异性肿瘤疫苗

NY-ESO-1也是一种肿瘤特异性抗原，在肺癌、黑色素瘤等多种恶性肿瘤有高表达。NY-ESO-1f（NY-ESO-1 91-110）肽疫苗，目前已结束Ⅰ期临床试验，10例入组患者中有2例肺癌患者及1例食管癌患者病情稳定，10例患者对于疫苗耐受良好，应用疫苗治疗后，9例（9/10）患者体内诱发出NY-ESO-1抗体反应，9例（9/10）患者体内CD4及CD8 T细胞反应增加^[20]^，其临床效果需进一步临床试验验证。

##### 2.1.1.5 belagenpumatucel-L

belagenpumatucel-L是一个肿瘤细胞疫苗，包括4种辐照后不同的NSCLC细胞系（2个腺癌，1个鳞癌，1个大细胞癌），可以呈递一系列的抗原，并用含TGF-β2的反义mRNA转基因质粒进行转染。设计抗TGF-β2的反义mRNA的表达以减少该细胞因子的产生，从而提高该疫苗的免疫原性。该疫苗的安全性在75例NSCLC患者中验证^[21]^，在Ⅱ期实验中，患者每月注射12.5×10^6^、25×10^6^或50×10^6^细胞，累及16次。在61例晚期患者（Ⅲb期和Ⅳ期）中，15%患者达到PR。试验发现，该疫苗相关的生存率与给药的细胞数相关，高剂量组（> 25×10^6^）患者2年生存率52%，低剂量组为20%。在61例晚期患者中进行免疫功能评估，细胞因子的增高（干扰素γ和IL-6）与有效的治疗反应相关。进一步研究发现，低的外周血肿瘤细胞的数目与较长的OS相关^[22]^。基于上述结果，该疫苗的Ⅲ期临床试验已开始进行。

##### 2.1.1.6 表皮生长因子（epidermal growth factor, EGF）疫苗

表皮生长因子受体（epidermal growth factor receptor, EGFR）信号通路是NSCLC治疗的一个重要靶通路，针对该信号通路的EGF疫苗，可以诱导体内产生抗EGF抗体^[23]^，进而起到抗肿瘤效果。EGF疫苗是古巴研制，是一个重组的人EGF疫苗。

在Ⅱ期临床实验中，应用EGF疫苗后73%患者血液中检查到很好的EGF抗体反应，58%患者可以有效阻断EGF/EGFR信号通路^[24]^。在Ⅱ期临床试验中，80例Ⅲb期或Ⅳ期完成一线化疗后患者随机应用EGF疫苗或BSC^[25]^。应用CTX后，50 ug当量的EGF疫苗每周应用4周后每月应用，治疗组可以看到延长生存期的倾向（治疗组6.5个月*vs*对照组5.3个月，*P*=0.098）。亚组分析可以发现，年龄低于60岁患者，明显获益（11.6个月*vs* 5.3个月，*P*=0.0124）。有效的免疫反应预示获益好，EGF抗体滴度大于1:4, 000，或者至少4倍于之前免疫效价的患者中位OS为11.7个月，其余的中位OS为3.6个月（*P*=0.002）。目前该疫苗已经在古巴上市，应用于Ⅲb期和Ⅳ期NSCLC患者，进一步的Ⅱ期/Ⅲ期临床试验在马来西亚进行。

##### 2.1.1.7 DC细胞疫苗

DC细胞是功能最强的抗原递呈细胞，具有独特的刺激T细胞增殖的能力，是机体免疫应答的始动者。抗原呈递在诱导抗肿瘤免疫应答中起关键作用，利用负载肿瘤抗原的DC进行恶性肿瘤的免疫治疗是一种抗肿瘤主动免疫治疗的研究方向。目前，已建立了多种方法制备基于DC的肿瘤疫苗，如用肿瘤细胞裂解物或凋亡的肿瘤细胞等冲击致敏DC、肿瘤相关抗原肽段直接致敏DC、将肿瘤相关抗原基因通过电穿孔或质粒DNA或病毒性载体直接转染DC等^[26]^。Um等^[27]^应用肿瘤裂解物致敏DC细胞作为肿瘤疫苗，应用于15例Ⅲ期或Ⅳ期NSCLC患者，患者耐受性良好，且两个肿瘤有缓解，提示对于DC细胞疫苗，患者可能有获益。

#### 2.1.2 被动免疫治疗

被动免疫治疗指用体外产生免疫效应器，输入患者体内，进而起到增强免疫治疗的效果。最常见的被动免疫的形式是注射重组细胞因子、免疫效应细胞、DC细胞和单克隆抗体，因为直接针对肿瘤的单克隆抗体为靶向治疗的重要组成部分，在此不赘述。

##### 2.1.2.1 细胞因子

细胞因子在宿主抗肿瘤的自身免疫中发挥重要的作用，例如，干扰素（interferon, IFN）γ和IL-2是主要的淋巴细胞生长因子，可以扩增细胞因子诱导的杀伤细胞（cytokine-induced killer, CIK），TNFα一个激活免疫治疗的有效佐剂中。在NSCLC免疫治疗中，IL-2发挥一定的作用，研究最多的是IL-2。然而，临床研究表明，IL-2联合化疗，与单纯化疗组相比较，1年OS和中位PFS均无优势^[28]^。重组IL-2联合PBMC治疗NSCLC^[29]^，虽然5年OS有提高（59% *vs* 32%），但对于整体OS及无时间生存时间（event-free survival, EFS）均无统计学差异。

##### 2.1.2.2 过继免疫治疗

过继免疫是把致敏淋巴细胞输给肿瘤患者，使其获得抗肿瘤免疫力。过继免疫治疗最早用于血液病骨髓移植后治疗，后来逐渐用于实体瘤的治疗，并有一定的临床效果。Zheng等^[30]^回顾总结了近年来应用于NSCLC的不同的过继细胞方法：淋巴因子激活的杀伤细胞（lymphokine-activated killer, LAK）是由淋巴细胞暴露于IL-2衍生而得，可以破坏NK细胞耐受的肿瘤；肿瘤浸润淋巴细胞（tumor-infiltrating lymphocytes, TIL）有针对肿瘤抗原的高亲和力T细胞受体，可以识别各种明确的和不明确的肿瘤抗原，TIL可以在体外有效扩增，并可探及对肿瘤的反应，不幸的是，TIL需要通过手术或活检取得，使得其应用受到局限；CIK细胞可以通过体外应用抗CD3单克隆抗体、IL-2、IL-1α和IFNγ作用于外周血淋巴细胞孵化而得，CIK细胞具有多种T淋巴细胞和混合的T-NK表型，具有不受MHC限制的抗肿瘤活性；γ δ T细胞在外周血CD3^+^T细胞中仅占1%-10%，其可以在无MHC情况下识别抗原，作为一种APC细胞直接影响免疫反应，动物实验显示γ δT细胞有保护性的免疫监视和治疗性的抗肿瘤活性；肿瘤相关抗原（tumor associated antigen, TAA）特异性的细胞毒性T细胞（cytotoxic T-cell, CTL）是通过外周血单个核细胞在体外由特异性的肿瘤抗原肽刺激产生，可以杀死表达这个肽的肿瘤细胞。

大量临床试验显示，过继免疫治疗联合手术治疗、放化疗有一定的临床疗效，但对延长PFS、OS无确切的临床试验证据。此外，过继免疫治疗的局限性也限制其发展：首先，细胞培养的费用很高，不是每位患者都能承受，过程也是个体化的，不能像药物可以批量生产。第二，免疫反应需要长时间的评估，而且需要足够数目的细胞。第三，获得这些免疫细胞过程复杂，条件要求高，对于有感染，外周血管疾病或高血压的患者都不能有效获得PBMC的收集。

##### 2.1.2.3 DC细胞联合过继免疫治疗

目前DC细胞联合其他过继免疫细胞协同抗肿瘤治疗也是肿瘤免疫治疗的一个发展方向。Zhong等^[31]^进行的前瞻性临床试验，DC细胞/CIK细胞联合化疗用于NSCLC治疗，每30天接受两次以上免疫治疗组患者较每30天接受两次免疫治疗的患者，肿瘤进展时间（time-to-progression, TTP）延长（7.3个月*vs* 6.2个月，*P*=0.034）。该小组的另一个研究显示，相对于单纯化疗组，DC细胞/CIK细胞联合化疗组患者TTP延长（6.9个月*vs* 5.2个月，*P*=0.034），但OS无延长（*P*=0.18）。Li等^[32]^研究发现Ⅰ期-Ⅲa期术后患者应用DC细胞/CIK细胞免疫治疗联合化疗组，相对于单纯化疗组，两年生存率提高（94.7%±3.6% *vs* 78.8%±7.0%, *P* < 0.05），但两年DFS无统计学差异。

#### 2.1.3 支持性免疫治疗

支持性免疫治疗是指通过非特异性的增加机体本身的免疫系统。人体乳转铁蛋白（lactoferrin）是一种铁绑定糖蛋白，可激活性淋巴激活杀伤细胞和自然杀伤细胞，加强多核巨细胞和巨噬细胞细胞毒性^[33]^。重组人体乳转铁蛋白（talactoferrin alpha; talactoferrin, TLF）是研究一种口服免疫调节剂，在最近的Ⅱ期临床实验^[34]^中，110例局部晚期或是转移的NSCLC初治患者，接受CP的化疗的同时接受TLF或安慰剂，在100例可评价的患者中实验组患者有效率是47%而对照组是29%（*P*=0.05），OS也有延长（11.3个月*vs* 8.5个月，HR=0.75，*P*=0.11），此外治疗组副作用更少（总副反应：472 *vs* 569，*P*=0.003）。可能是由于TLF增加了免疫系统的重建。目前两个Ⅲ期临床试验正在进行。

### 2.2 免疫靶向治疗

靶向治疗针对性强，副作用少，成为肿瘤治疗的新宠，对于肿瘤免疫治疗，科学家们也想找到相应的靶点，特异性的调节抗肿瘤免疫功能。肿瘤细胞和周围基质造成的环境可以使T细胞在数目和活性方面受损，对肿瘤生长有利，这种由肿瘤或肿瘤相应周围细胞产生的免疫抑制，已经成为肿瘤的特点。T细胞活性的关闭有几个免疫相关的检查点，其中细胞毒性T淋巴细胞相关抗原4（cytotoxic T lymphocyte antigen-4, CTLA-4），T细胞免疫球蛋白粘蛋白3和程序死亡-1（programmed death-1, PD-1）是调节T细胞聚集和效用功能的共阻碍因子，与他们的配体相结合可导致T细胞对于肿瘤细胞的效应减弱，持续的信号可导致T细胞功能耗竭，包括分化、分泌细胞因子、裂解肿瘤细胞的功能都相继失去。通过改变T细胞的免疫抑制，可以有效的提高免疫系统对于肿瘤的杀伤作用。T细胞活性检查点，成为免疫治疗的重要的靶点，目前针对于T细胞活性检查点，研究者开发了特异性的检查点靶向阻滞剂，在临床实验中逐渐显示出有效的抗肿瘤治疗效果。

#### 2.2.1 Ipilimumab

T细胞被激活成为效应细胞的早期，T细胞表面诱导表达CTLA-4，CTLA-4可绑定于APC的共刺激分子CD80、CD86。通过CTLA-4绑定于APC的CD80、CD86，T细胞被传递了负信号，T细胞被阻止进一步的功能活性，这些T细胞会通过凋亡消除^[35]^。针对CTLA-4的抗体可以阻止这个途径，进而增强T细胞免疫应答，增加抗肿瘤的免疫活性。Ipilimumab是一个抗CTLA-4的人单克隆抗体，可以有效阻断CTLA-4相关T细胞活性抑制信号，使得效应T细胞在肿瘤内增长和浸润，导致肿瘤细胞坏死。

Ipilimumab联合化疗治疗晚期NSCLC的Ⅱ期临床试验^[36]^，一共204例患者入组，按照1:1:1的比例分为CP方案组（卡铂+紫杉醇）同步给予ipilimumab组（前4周期每次给予ipilimumab，后2周期给予安慰剂）、CP方案序贯ipilimumab组（前2周期给予安慰剂，后4周期给予ipilimumab）及对照组CP方案组，有治疗效果的患者每12周给予ipilimumab或安慰剂以作为维持治疗，对于治疗效果的评估分别采用免疫相关有效率评估标准^[37]^和WHO标准。结果显示：化疗序贯应用ipilimumab组患者免疫相关无疾病进展时间（immune-related PFS, irPFS）有实质性改善（序贯组*vs*对照组=5.7个月*vs* 4.6个月，HR=0.72，*P*=0.05），序贯应用组也延长了WHO标准的PFS（序贯组*vs*对照组=5.1个月*vs* 4.2个月，HR=0.69，*P*=0.02），并且有延长OS的趋势（序贯组*vs*对照组=12.2个月*vs* 8.3个月，HR=0.87，*P*=0.23），而同步应用组无统计学差异。进一步分析提示，尽管无明显统计学差异，序贯应用ipilimumab更利于达到临床效果，在肺鳞癌患者胜于非鳞癌患者。Ⅲ期临床研究ipilimumab联合CP方案的治疗Ⅳ期肺鳞癌患者正在进行，计划纳入920例患者，进一步评估该治疗方案的有效性。广泛期小细胞肺癌（extensive-disease small-cell-lung cancer, ED-SCLC）患者应用ipilimumab也有明显的irPFS效果（治疗组*vs*对照组=6.4个月*vs* 5.3个月，HR=0.64，*P*=0.03）^[38]^，可能对于ED-SCLC患者有效。Ipilimumab联合EP治疗ED-SCLC正在进行Ⅱ期临床试验，Ⅲ期临床试验也计划进行。

#### 2.2.2 BMS-936558和BMS-9365589

免疫耐受的一种机制是肿瘤通过PD-1途径启动抑制路径。PD1（CD279）是B7-CD28家族中的一员，是一个细胞表面的受体，有两个配体：PD-L1（B7-H1）和PD-L2（B7-DC）。PD1在活性CD4^+^和CD8^+^ T、NK、B、激活的单核细胞和成熟的DC细胞表达。通过激活PD-1信号通路，T细胞可触发凋亡。PD-1途径是一个重要的调节因子，可以诱导和维持免疫耐受，保护组织不受自身免疫的伤害^[39]^。在NSCLC患者观察到PD-1在CD8^+^T细胞过表达，PD1^+^CD8^+^T细胞减弱生产细胞因子和扩增的能力，此外，发现NSCLC患者肿瘤中PD-L1阳性细胞明显高于肿瘤旁肺组织^[40]^，PD-L1在肺癌细胞的表达与预后差相关。因此假设封闭PD1途径可以修复和促进“衰竭的”肿瘤特异性T细胞的功能，进而减少肿瘤诱发的免疫抑制。抗PD抗体lambrolizumab（BMS-936558）和抗PD-1-L1抗体BMS-936559已进入临床研究阶段。BMS-936558的Ⅰ期临床实验^[41]^共纳入296例实体瘤患者，接受8周的治疗。结果发现，NSCLC患者中，18%（14/76）有治疗反应。基于上述结果，考虑PD-1信号通路抑制剂在NSCLC治疗方面存在潜力，进一步的临床研究正在进行。

## 3 免疫治疗的抗肿瘤疗效评估

随着免疫治疗越来越多的应用于实体肿瘤的治疗，针对抗肿瘤的细胞毒药物疗效评估的RECIST标准^[42]^和传统的WHO标准，并不能很好的评估免疫治疗在肿瘤治疗中的疗效。在免疫治疗中，如果疗效评估为稳定（stable disease, SD），提示是一种有意义的治疗效果，而且即使经免疫治疗后RECIST评估为疾病进展（progressive disease, PD），随后可能转变为完全缓解（complete response, CR）、部分缓解（partial remission, PR）或SD。肿瘤学家、免疫学家们总结免疫治疗特点得出共识^[43]^：①肿瘤免疫治疗出现可测量的抗肿瘤活性的时间比细胞毒性药物出现的时间长；②治疗反应可能出现在常规评估的PD之后；③除非已经确定PD，停用免疫治疗是不合适的；④临床上轻微的PD是可接受的；⑤持续的SD可能代表了抗肿瘤效果。基于上述思想，制定出了实体瘤免疫治疗疗效评估指南－免疫治疗疗效评估标准（immune-related response criteria, irRC）^[37]^，在新的指南中，抗肿瘤疗效基于所有可测量病灶（每个脏器10个病灶或5个皮肤病灶）的肿瘤两个最大垂直径乘积之和（sum of products of the two greatest perpendicular diameters, SPD），得出总肿瘤负荷进行评估，新发病灶计入总肿瘤负荷进行评估。irRC标准与传统的WHO标准相比较^[37]^：如新发现可测量病灶（≥5 mm×5 mm），WHO标准永远代表PD，而irRC标准中需要纳入总肿瘤负荷再评价是否PD；如新发现不可测量病灶（如 < 5 mm×5 mm)，WHO标准永远代表PD，而irRC标准不定义为进展（但排除irRC）；对于PR，WHO标准需在至少间隔4周的两次连续的观察点均证实SPD较基线下降≥50%且未见新发病灶或其他病变进展，irRC标准中仅需在至少间隔4周的两次连续的观察点均证实总肿瘤负荷较基线肿瘤负荷下降≥50%；对于SD，WHO标准需在两次连续的观察点检测到SPD较基线下降 < 50%或SPD增大 < 25%且未见新发病灶或其他病变进展，irRC标准仅需在两次连续的观察点证实总肿瘤负荷较基线肿瘤负荷下降 < 50%或增加 < 25%；对于PD，WHO标准在任一观察点检测到SPD较基线增加≥25%和（或）出现新发病灶和（或）出现其他病变进展，而irRC标准在至少间隔4周的两次连续观察点的任一时间检测到总肿瘤负荷较基线肿瘤负荷增加≥25%。irRC在ipilimumab治疗黑色素瘤Ⅱ期临床试验结果中进行评估，验证了该标准的合理性和可行性，可以对肿瘤的免疫治疗效果做出更加准确和客观的评价。

## 4 总结

综上所述，NSCLC的免疫治疗方法不断发展，出现了肿瘤疫苗、检查点阻滞剂等多种新型抗肿瘤药物，并在初期临床试验中看到一定的成果，新的免疫治疗疗效评估标准为更好的评估肿瘤免疫治疗效果奠定了基础。目前NSCLC的免疫治疗已成为继手术、化疗、放疗和靶向治疗之后的第五种重要的治疗方法，很好地了解NSCLC免疫逃逸基础和免疫治疗方法，进一步指导NSCLC患者临床治疗方案选择，延长NSCLC患者生存期有重要意义。
